# Functional Neurological Disorder with functional ptosis: Clinical challenges and opportunities for learning. (Case study)

**DOI:** 10.1192/j.eurpsy.2025.1225

**Published:** 2025-08-26

**Authors:** S. Crowley, O. O’Toole, E. O’Neill, J. Malaka, E. Kelleher

**Affiliations:** 1 South Lee Mental Health Services, Cork University Hospital; 2 Department of Neurology, Cork University Hospital, Wilton; 3 Department Of Psychiatry; 4 Department Of Consult Liaison Psychiatry, South Lee Mental Health Services, Cork University Hospital; 5 Department Of Psychiatry, University College Cork, Cork, Ireland

## Abstract

**Introduction:**

Functional Neurological Disorder (FND) is a well-recognised disorder that is seen by both neurology and psychiatry services. It is an unconscious disorder that may involve motor or sensory neurological symptoms, classically ascribed to underlying psychological distress, the aetiology of which can sometimes be difficult to ascertain.

**Objectives:**

This case presented highlights several key features of FND.

Presentations can involve pain and may evolve to involve multiple locations that may not follow a neuroanatomical distribution.

There may be initial reluctance of the patient or family to accept the diagnosis.

These can be difficult cases but there is potential for improvement with multidisciplinary support.

Inclusion of the patients’ perspective of going through the diagnostic process, in this case, gives a unique perspective of being a patient with this disorder,

**Methods:**

.

**Results:**

A young female presented to the emergency department with acute onset severe right facial pain, subjective facial swelling, minor ptosis of the right eyelid and headache. Symptoms evolved over the next week to include speech disturbance, right shoulder pain, and reduced sensation in her right arm. After initial extensive negative work, it was felt that the patient most likely had a functional neurological disorder and underwent multidisciplinary treatment. The patient represented four months later after a minor fall at work with re-emergence of the right arm and facial pain associated with speech disturbance. Relapsed functional neurological disorder (FND) was diagnosed. A multidisciplinary team (MDT) approach with neurology, liaison psychiatry, speech and language therapy (SALT), physiotherapy and psychotherapy was instituted resulting in the resolution of symptoms. FND is a common disorder in which clinicians receive limited training. This case highlights a complex presentation of FND including “pseudoptosis”, a rarely seen functional symptom, and how MDT input led to symptomatic improvement. Relapse of FND is not uncommon, sometimes after minor physical or psychological stress or trauma. There is hope to improve symptoms when this happens. Our review also includes the patient’s perspective of going through the FND diagnosis.

**Conclusions:**

Functional Neurological Disorders are common and greater training in and understanding of these disorders is important.

Symptoms that are rarely functional such as ptosis, do not exclude FND.

FND can be a difficult diagnosis for patients and families to accept, but accepting the diagnosis is key to appropriate treatment and recovery

An MDT approach incorporating neurology, psychiatry, physiotherapy, psychology, speech and language therapy and occupational therapy provides the best opportunity for recovery.

Early diagnosis and multi-disciplinary treatment can aid recovery, reduce the development of further disability and reduce healthcare utilisation and costs.

**Disclosure of Interest:**

None Declared

